# Soft, Conductive, Brain-Like, Coatings at Tips of Microelectrodes Improve Electrical Stability under Chronic, In Vivo Conditions

**DOI:** 10.3390/mi12070761

**Published:** 2021-06-28

**Authors:** Arati Sridharan, Jit Muthuswamy

**Affiliations:** School of Biological and Health Systems Engineering, Ira A. Fulton School of Engineering, Arizona State University, Tempe, AZ 85287-9709, USA; arati.sridharan@asu.edu

**Keywords:** brain implants, neural prostheses, PDMS, silicone, chronic implants

## Abstract

Several recent studies have reported improved histological and electrophysiological outcomes with soft neural interfaces that have elastic moduli ranging from 10 s of kPa to hundreds of MPa. However, many of these soft interfaces use custom fabrication processes. We test the hypothesis that a readily adoptable fabrication process for only coating the tips of microelectrodes with soft brain-like (elastic modulus of ~5 kPa) material improves the long-term electrical performance of neural interfaces. Conventional tungsten microelectrodes (*n* = 9 with soft coatings and *n* = 6 uncoated controls) and Pt/Ir microelectrodes (*n* = 16 with soft coatings) were implanted in six animals for durations ranging from 5 weeks to over 1 year in a subset of rats. Electrochemical impedance spectroscopy was used to assess the quality of the brain tissue–electrode interface under chronic conditions. Neural recordings were assessed for unit activity and signal quality. Electrodes with soft, silicone coatings showed relatively stable electrical impedance characteristics over 6 weeks to >1 year compared to the uncoated control electrodes. Single unit activity recorded by coated electrodes showed larger peak-to-peak amplitudes and increased number of detectable neurons compared to uncoated controls over 6–7 weeks. We demonstrate the feasibility of using a readily translatable process to create brain-like soft interfaces that can potentially overcome variable performance associated with chronic rigid neural interfaces.

## 1. Introduction

Stability in the long-term performance of neural implants remains an elusive goal. It is widely shown that the signal reliability of chronic recordings from neural probes tend to fail within a few weeks to several months after implantation [1]. It has been shown that the mean failure time of implanted silicon microelectrode arrays (MEAs) in non-human primate is 334 days [2]. Implant failure, especially under chronic conditions, is likely due to multiple abiotic (i.e., probe corrosion and insulation failure) and biotic factors (i.e., loss of neurons at the interface, gliosis and oxidative stress due to neuroinflammation, etc.) [3,4,5,6,7,8,9,10,11,12]. Initial penetration of the probes into brain tissue induces tissue shearing that propagates inflammation and oxidative stress, which are hypothesized to result in a source of implant failure [13]. Various studies support that mitigation of oxidative molecules at the interface using either anti-inflammatory components or miniaturization of the electrode surface using ultra-small probes can improve biocompatibility [14,15,16,17,18,19,20,21,22,23].

Under chronic long-term implantation conditions, a source of continuous mechanical stress at the interface is hypothesized to be induced by physiological tissue micromotion due to movement, respiration and vascular pulsations [24,25]. Brain tissues at the stationary electrode–brain interface are known to undergo significant changes in the mechanical properties (such as elastic moduli) over a period of 4–8 weeks in response to a stiff implant and micromotion induced stresses correspondingly increase at the interface [25]. The continuous chafing between tissue and stiff electrode at the interface is hypothesized to trigger inflammation under chronic conditions [26]. Design strategies to reduce the mechanical mismatch, including fabrication of probe substrates using flexible and compliant materials (thermoplastics, hydrogels, elastomers, etc. [27,28,29,30,31,32,33,34,35,36,37,38,39,40,41]), have shown improvement in biocompatibility. An emerging view is that the reduction in glial scarring and immune response to ‘soft’ implants due to reduced mechanical mismatch is mediated through significantly improved strain rates on the brain tissue at the interface [42].

Finite element models of the brain electrode tissue interface suggest that the maximum stresses typically occur at sharp edges of the electrode trace and the probe tip, where the recording electrode sites are typically located in many types of arrays [43,44,45,46,47]. The functional performance of the neural probes, which is determined by its electrical properties (i.e., electrode impedance, signal characteristics such as peak-to-peak voltage (Vpp) of isolated units and signal-to-noise ratios (SNR) at the interface) have shown dynamic changes over a duration of 21 weeks in rats [7,8,9,10], often resulting in failure for various implanted microarrays (tungsten, platinum-iridium and silicon) [7,8,9,48]. We previously showed that soft brain-like elastomer coatings on stiff carbon fiber-based probes significantly reduce stress at the tissue interface [49]. In this study, we hypothesize that coating of electrode recording sites with a soft brain-like (elastic modulus (E)~5–8 kPa), conductive elastomer that is closely matched with the viscoelastic properties of the brain will improve the long-term electrical performance of conventional microwire arrays.

Recent studies that have reported the use of elastomers and other soft neural interfaces in chronic experiments required custom fabrication processes [27,28,31,34,35,36,37,39,40,41]. We report here a post-processing technique that can be readily translated to a wide variety of current microelectrodes, such as the Utah recording array with recording sites located at the tip used in animal studies and clinical applications [48]. The post-processing technique tested and reported in this study involves a dip-coating procedure that is readily adoptable by laboratories across the world.

## 2. Materials and Methods

### 2.1. Fabrication of Soft Elastomeric Interface

Development of the soft conductive elastomeric interface is briefly reported elsewhere [49]. Briefly, polydimethylsiloxane (PDMS) precursors (Sylgard 184 elastomer kit, Dow Corning) were used to fabricate the soft elastomeric composites by varying the ratio of base-to-crosslinker. Sylgard 184™ has been shown in various studies for implants in vivo to be biocompatible [50,51]. The soft elastomeric substrate was incorporated with carbon nanotubes (Sigma-652490-carboxylic acid functionalized, single-walled carbon nanotubes (CNT)) for enhanced conductivity at < 1% *w*/*v*. The viscoelastic properties of the resultant elastomer composite with carbon nanotubes were matched with the viscoelastic properties of rodent brain tissue that are in vivo determined in a prior study where we had shown that pristine cortical brain tissue had typical elastic moduli of around 3–10 kPa with shear moduli around 1.4–3 kPa [25]. The mixture was rested for at least 20 min under vacuum to get rid of bubbles, after which it was dip-coated on the recording sites of tungsten or platinum-iridium microwire electrodes (Microprobes, Inc., Gaithersburg, MD, USA). In order to coat the electrode tips on the array, a stereotaxic frame with 10 µm resolution was used to mount the MEA and lowered using the manual z-axis micromanipulator into the premix ~150 µm to cover the tip completely. The MEA tips were placed in the premix for 30 s to dip coat the probes and subsequently the probes were withdrawn from the mixture slowly by manual retraction of the micromanipulator. Excess premix was removed gently using Whatman filter paper (Grade 1). The coated electrodes were placed in a dry incubator (60 °C) with low humidity (<5%) for 18 h after which it remained well-adhered to the underlying probe surface. The soft, elastomeric composite with CNT had water contact angles of 97–103° based on goniometry measurements determined by automated instrument software (Ramé-Hart, Netcong, NJ, USA). The coated probes were rinsed in deionized water three times and passivated by storing them in artificial cerebrospinal fluid (aCSF) (7.4 g sodium chloride, 2.1 g sodium bicarbonate, 0.17 g sodium phosphate monobasic, 0.19 g magnesium chloride and 4.5 g glucose in filtered deionized water) at least 24 h prior to implantation to prevent hydrophobic recovery at the interface. The resistivity (ρ) of the final crosslinked brain-like coating was estimated to be 31.0–41.3 kΩ-cm. Prior to treatments, the probes were dipped in 70% ethanol for 20 min. The probes with the coatings were UV sterilized and assembled in a biosafety cabinet (BSL2) and stored in 0.2 µm filtered aCSF.

### 2.2. Mechanical Characterization

Microindentation was used to characterize the mechanical properties of soft, brain-like composites as described elsewhere [49]. Briefly, bulk samples of soft, brain-like silicone composites were fabricated in 2.5 cm diameter with a depth of 0.5 cm flat bottom wells and indented 200 µm using a stainless-steel spherical indenter (4 mm diameter). Force was measured using a 10 g load cell (Futek Inc., Irvine, CA, USA). The values for elastic moduli were calculated using a Hertzian model, where the elastic moduli were estimated using the following standard equation for spherical indenters:

F = (4/3) (E/1 − ν^2^) (√R) (δ^(3/2)^) (1)

where F is the measured force, E is the elastic modulus, ν is the Poisson’s ratio assumed to be 0.5 for silicone based elastomeric materials, R is the radius of the and δ represents the indentation depth. The elastic modulus of the final soft brain-like silicone-CNT composite was estimated to be 5 ± 3 kPa using the microindentation technique.

For assessing long-term mechanical stability, samples were placed in aCSF for at least 8 weeks inside an incubator at 37 °C, 95–99% humidity and 5% CO_2_ to simulate body conditions. After 4 weeks, a subset of samples (*n* = 3) was taken out and measured for change in elasticity and swelling based on gravimetric measurements. No statistical significance was found in the elastic modulus between day 1 (4.2 ± 1.3 kPa) and 4 weeks (2.1 ± 0.8 kPa) using Student’s *t*-test (α = 0.05 criterion). An additional set of samples (*n* = 3) was used for measuring contact angles after 8 weeks.

Step indentation protocols were used for the accurate comparison of material properties of the elastomer/hydrogel to in vivo cortical values at differential depths due to the small strains experienced by implants (mainly micromotion) and for characterizing the brain and soft silicone composite [25,42,49]. Viscoelastic material relaxation properties using the penetrating microindentation technique [49] were compared for 3 groups that include the following: 1) cortical brain tissue (*n* = 4 animals); (2) soft brain-like silicone/CNT composites (*n* = 4 samples); and (3) 0.5% agarose hydrogels (*n* = 3 samples) composed of 0.5% (*w*/*v*) agarose (CAS#9012-36-6) and 0.9% saline. The in vivo data for sample group (1) for rat cortical brain tissue were taken from [25], which used the same protocol used for sample group (2) and (3). Agarose hydrogels are widely used as brain phantoms [52,53,54]. Briefly, a stainless steel conical probe was used to penetrate the material and the material relaxation forces imposed on the probe were measured using a 10 g load cell at a sampling frequency of 54 Hz. The relaxation force curves obtained from the load cell were normalized and fitted to a second order Prony series model using the MATLAB curve fitting toolbox and the long-term (not shown in this study) and short-term relaxation time constants were calculated. No significant differences were observed among the long-term relaxation time constants for the 3 groups (not shown here). The statistical mean and standard deviations for the short-term relaxation time constants, which are implicated in strain rates, are reported in this study. One-way ANOVA showed statistically significant differences among the three groups (*p* < 0.05). Statistical comparisons between agarose and soft silicone/CNT composites were performed using Student’s *t*-test with an α criterion of 0.05.

### 2.3. FTIR Characterization

Soft brain-like silicone composites were fabricated and coated on a silicon (100) wafer substrate. In order to characterize the molecular structures on the surface of the novel silicone-CNT composite, the Fourier transform infrared with attenuated total reflection (FTIR-ATR) spectrum was acquired for each material using a Bruker IFS 66V/S IR spectrometer with a wide-band mercury cadmium telluride (MCT) detector cooled by liquid nitrogen under vacuum (at the Center for Solid State Science at Arizona State University. CSSS-ASU). The spectrometer was equipped with a germanium ATR crystal (Harrick ATR-GATR); the resolution was 4 cm^−1^. One-hundred and twenty-eight scans were generated and averaged for *n* = 3 samples.

### 2.4. Cell Culture Experiments

Primary cortical neurons (E18 mice) were purchased from BrainBits, LLC (Springfield, IL, USA) and seeded at 5000 cells/ mm^2^ on soft and ‘hard’ silicone/CNT composite substrate (*n* = 4 of each type). Soft (~5 kPa) and hard (0.300–1 MPa) silicone/CNT composite substrates were made by varying the crosslinker-to-base ratio. Substrates were pre-coated with 1 µg/mL polyethyleneimine (PEI) for 6 h, washed thrice with sterile distilled water and dried overnight. The neurons were cultured at 37 °C and 5% CO_2_ in NbActiv1 media (BrainBits) for DIV 7 (i.e., 7 days in vitro) and subsequently imaged after 7 days after incubation with Calcein AM (Sigma) as a live cell marker. Live cells were counted on each type of soft and hard substrate manually by using the cell counting feature in the Fiji/ImageJ software [55] and the statistical mean and standard deviation were calculated. Neurites emanating from the cell body were defined as primary, while neurites emanating from primary branches were defined as secondary branches. For enriched glia studies, substrates were pre-coated with a collagen-based cell attachment factor and passaged at least three times in a DMEM based medium (Cell Applications) to remove neurons. Cells were seeded on soft and hard substrates (*n* = 4 each) for 36 h and imaged using Mitotracker CMXRos™ as a live cell marker. Live cells were counted on each type of soft and hard substrate and the statistical mean and standard deviation were calculated. Statistical comparisons using Student’s *t*-test were performed with an α criterion equal to 0.05.

### 2.5. Animal Surgery and Implantation

Customized tungsten arrays (Microprobes, LLC., Gaithersburg, MD, USA) with 3 probes (125 µm diameter shaft that tapered down to ~7–10 µm near the exposed recording tip) that were spaced 1 mm apart were used in this study. The total length of the probes was 5 mm. A total of 9 tungsten electrodes were coated at the tip with the soft brain-like silicone composite and a total of 6 uncoated tungsten electrodes were used as negative controls. The exposed recording tips were typically 45 to 75 µm in length based on the manufacturer’s specifications. The thickness of the coating at the tip was ~20–40 µm and covered the recording site entirely. All *n* = 5 animals were implanted with the tungsten arrays in the somatosensory region. In addition, one more animal was implanted with a 16-channel-coated platinum–iridium (Pt/Ir) electrode array in the somatosensory region, including the barrel cortex region. Probes for the Pt/Ir MEA were placed 0.5 mm apart in a 4 × 4 format. The probes were 5 mm long with 75 µm shaft diameter that tapered to ~7–10 µm near the exposed recording tip. Similar to tungsten probes, the exposed recording tips were typically 45 to 75 µm in length based on the manufacturer’s specifications. Table 1 shows the cohort of animals with details on the length of implantation and the number of coated and uncoated electrodes in each array. All animal procedures were conducted with the approval of the Institute of Animal Care and Use Committee (IACUC) of Arizona State University and in accordance with the National Institute of Health (NIH) guidelines. All efforts were made to minimize animal suffering and to use only the number of animals necessary to produce reliable scientific data.

The surgical implantation procedure has been adapted from a previously described protocol [25]. Briefly, adult CD rats were induced using 50 mg mL^−1^ ketamine, 5 mg mL^−1^ xylazine and 1 mg mL^−1^ acepromazine administered via intraperitoneal injection. The anesthesia for the update contained a mixture of 50 mg mL^−1^ ketamine and 5 mg mL^−1^ xylazine and were injected based on the toe-pinch test. Rats received the analgesic of buprenorphine (0.05 mg kg^−1^) every 12 h for 48 h after surgery was completed. In order to implant the array, the head of the rat was shaved and mounted onto a stereotaxic frame (Kopf Instruments, Tujunga, CA, USA). After the skull was exposed, six stainless-steel bone screws (19010-10 Fine Science Inc., Foster City, CA, USA) were screwed into the skull to act as anchors, of which two of the screws were also used as grounds (~2–3 mm anterior to bregma and ~2.5 lateral to midline with one screw in each hemisphere). One craniotomy (~3.0 mm in diameter) was drilled in the right somatosensory cortex with the center point being 2.5 mm lateral to midline and 2.5 mm posterior to bregma. The dura was incised to allow for microarray insertion. The tungsten microelectrode array was slowly inserted into the brain at a rate of 10 µm s^−1^ and was implanted to a depth of 1.4 mm in the rodent somatosensory cortex for all chronic experiments. The positioning and orientation of the electrode array within the craniotomy was such that one electrode (1) was close to the midline, while electrode (3) was 1 mm contralateral to (1) and electrode (2) was 1 mm caudal to (1). The reference electrode was 1 mm ipsilateral to (1). The ground and reference were shorted. For the 16-channel platinum–iridium array that was dip-coated according to the procedure outlined in Section 2.1, the craniotomy (~5.0 mm diameter) was made in the left somatosensory region centered at 3.0 mm lateral to the midline and 2.4 mm posterior to the bregma. The individual electrodes were spaced 0.5 mm apart within the array as seen in Figure 10. The array was inserted at a 15° angle to approach the barrel cortex (~1.0–1.5 mm depth) until whisker stimulated responses were evident in some channels of the array. After the stainless steel grounding wire was connected to two of the bone screws, gelfoam was placed around the microelectrode array over any exposed tissue. After implantation, dental cement (PMMA) was used to secure the array onto the skull.

### 2.6. Electrochemical Impedance Spectroscopy (EIS)

Broad-band EIS (1–100,000 Hz) was conducted using a 5 mV amplitude sine wave input signal with CH-660 electrochemical station (CH Instruments, Austin, TX, USA) and measurements were used to test for changes in impedance due to tissue remodeling at the electrode–tissue interface. The measurements represented a 2-electrode system with a working electrode and a stainless steel ground shorted with the reference. EIS is a widely used test to assess the integrity of neural interfaces, where changes in impedance spectra are sensitive to both biotic (molecular and cellular changes in the surrounding tissue) and abiotic (material changes in the implant conductors and insulation, etc.) mechanisms at the interface [8,9,56,57]. Measurements were performed once a week for 5–7 weeks in all animals, at 6 months and 435/437 days for *n* = 2 animals. For the coated platinum–iridium array implanted in the rat barrel cortex, measurements were performed at 1 day, 14 day and 140 day in one animal. At least three readings were taken at each time point and averaged and complex impedance spectra (Nyquist diagrams) for all the electrodes were plotted. Impedance measurements at 1 kHz were averaged for each coated and uncoated electrode at each time point and the mean and standard deviation were plotted. The 95% confidence intervals were calculated across all time points for coated and uncoated electrodes and plotted on respective graphs.

### 2.7. Neural Recordings

Neural recordings were taken from awake animals using a multi-channel recording system (TDT Inc., Alachua, FL, USA). Recordings were taken once a week for 5–7 weeks for *n* = 5 rats and at 6 months and at 432/437 days for *n* = 2 rats. For the coated platinum-iridium array implanted in the barrel cortex, recordings were taken at 1 week, 4 months and 5 months post implantation. Each recording session was ~30 min. The neural signals were sampled at 24.414 kHz and bandpass filtered from 300–3000 Hz, with a gain of 10,000. Spikes were sorted offline using a custom MATLAB program that utilized principal component analysis (PCA) and k-means sorting algorithm. For spike sorting, the signal was detected by extracting amplitudes that exceeded 3.5 times the standard deviation (SD) of the amplitude distribution after removal of movement artifacts. Signal to noise ratio (SNR) and the average noise floor level were determined at 1 s intervals using a MATLAB program. Noise was defined as all data points below the threshold (3.5 SD of raw data). Noise levels were calculated as twice the standard deviation of the noise data. The mean peak-to-peak amplitudes of the largest isolated single unit (Vpp) for different time points were calculated from artifact free raw signals. Similar to Ludwig et al. [58], SNR was determined by the ratio of the Vpp to twice the standard deviation of the noise data after the removal of signal data, as is described below.

SNR ~ Vpp/2× SD_noise_ (2)

### 2.8. Statistical Methods

For all experiments, the mean and standard deviations were calculated for at least *n* = 3 samples for in vitro and material characterization experiments. Overall, data was assumed to be normal since the data distribution in box plots were generally symmetric about the median and the mean and median were close in value. One-way ANOVA was performed for statistical comparison of three groups with an α criterion of 0.05 between brain, agarose and soft silicone/CNT composites followed by Student’s *t*-tests with Bonferroni correction (*p* < 0.016).

For in vivo experiments, coated and uncoated electrodes were pooled separately after implantation in 5 animals. The number of coated tungsten electrodes was *n* = 9 and coated Pt/Ir electrodes was *n* = 16, while the number of uncoated tungsten electrodes was *n* = 6. Statistical comparisons were performed using Student’s *t*-test with an α criterion equal to 0.05. In order to test for changes in single unit amplitudes for the Pt/Ir array, one-way ANOVA was used (α~0.05).

## 3. Results

### 3.1. Soft Brain-Like Coatings from Silicone/CNT Composites

**Figure 1:**
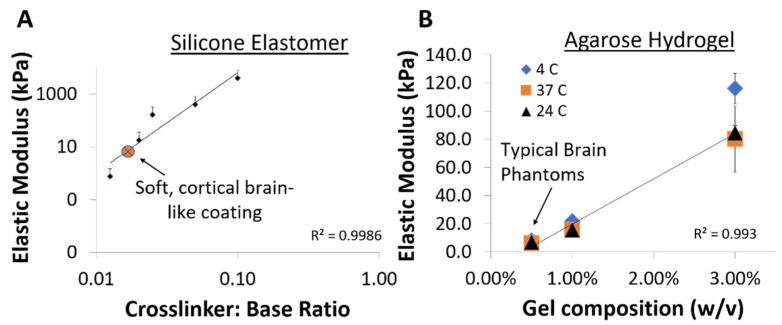
Estimated elastic moduli for various compositions of (**A**) silicone/nanotube elastomer and (**B**) agarose-based hydrogel compositions. The stability of the agarose gel compositions did not significantly change between 4 °C and 37 °C. The gel and elastomer compositions that mostly closely match the elastic modulus of cortical brain tissue is shown with an arrow. Mean ± standard deviations are shown.

In this study, we developed a novel, soft composite material composed of a silicone-based material with single-walled carbon nanotubes (CNT) incorporated in the silicone base. Based on Figure 1A, the soft, silicone composite was tuned to match cortical brain with an elastic modulus of approximately 5 ± 3 kPa (*n* = 3 samples), which is similar to a prior study where we showed that pristine cortical brain tissue had typical elastic moduli of around 3–10 kPa with shear moduli around 1.4–3 kPa [25]. The elastic modulus of the composite material as a function of ratio of silicone base-to-crosslinker is compared to the elastic moduli of various agarose hydrogel compositions in Figure 1B. Typical agar-based brain phantoms have a 0.5% gel composition to resemble cortical brain elastic modulus.

**Figure 2:**
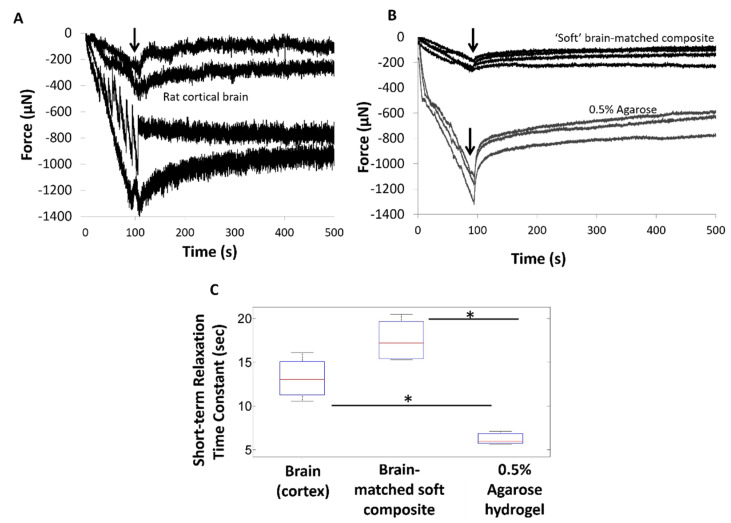
Typical force curves using a conical stainless steel probe in (**A**) rat cortical brain in vivo (data from [25]), (**B**) brain-like silicone/CNT composite and 0.5% agarose phantoms. Arrows indicate stoppage of probe movement with subsequent relaxation of brain tissue. (**C**) The short-term time constant of viscoelastic, relaxation phase of the force curves was determined using a second order Prony series model. Significant differences after correction by Bonferroni factor (* *p* < 0.016) in the short-term relaxation time constant were shown between in vivo brain, silicone/CNT composite and agarose hydrogel.

In order to compare in vivo mechanical characteristics with silicone gel compositions, the force-displacement curves using the penetrating micro-indentation test for cortical brain (*n* = 4 samples) are shown in Figure 2A and the corresponding curves for soft brain-like composite (*n* = 4 samples) and 0.5 % agarose hydrogel (*n* = 3 samples) (commonly used to model brain phantoms) are shown in Figure 2B. A stainless steel conical probe was moved to a depth of 1 mm at a constant speed of 10 µm /s in all of the above three materials (characterized by a monotonic increase in compressive force until it reached a maximum of 200–1400 µN), after which all materials exhibited viscoelastic relaxation (characterized by a monotonic decrease in compressive forces at different rates of relaxation). The force curves were similar in shape with increasing forces observed for both brain tissue and soft brain-like composite materials. Maximum forces of −61.3 ± −37.4 µN were observed during penetration in soft brain-like composites compared to maximum force values of −173.9 ± −137.3 µN in rodent cortical tissue. The maximum forces in soft brain-like composites were not significantly different from those in cortical brain tissue based on Student’s *t*-test (α = 0.05 criterion). Due to heterogeneity in material parameters, the peak compressive forces in the cortical brain tissue varied over a range and were comparable to those of both the agarose and the soft silicone composite substrates. The peak compressive forces in soft silicone composites were less than those in 0.5% agarose gel.

Viscoelastic characterization of the soft brain-like composite showed that the elastomeric material best fit a second order Prony series model typically used to model brain tissue under small strain condition [25]. The short-term relaxation time constants derived from the Prony series model was compared among rat cortical, soft silicone composite and the 0.5% agarose hydrogel as shown in Figure 2C. The short-term relaxation time constant for the brain tissue was found to be 13.2 ± 2.4 s. The soft brain-like composite had a short-term relaxation time constant of 17.5 ± 2.6 s (*n* = 4 samples), similar to the mean of rat cortical brain. Brain phantoms made of 0.5% agarose hydrogel were found to have a short-term relaxation time constant of 6.25 ± 0.77 s (*n* = 3 samples). One-way ANOVA showed statistically significant differences among the three groups (*p* < 0.05). Using Student’s *t*-test for pairwise multiple comparisons (after correction by the Bonferroni factor (*p* < 0.016), the short-term relaxation time constant for the agarose hydrogel was found to be significantly smaller compared to that of the soft, silicone composite (*p* = 0.0008) and the cortical brain tissue (*p* = 0.0055). However, the short-term relaxation time constants of cortical brain and soft silicone composite were not significantly different (*p* = 0.047).

**Figure 3:**
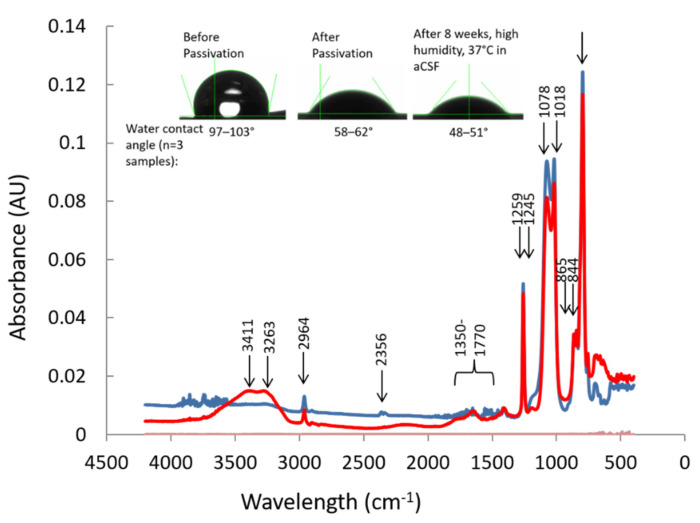
FTIR-ATR fingerprint regions of PDMS (blue) and soft brain-like composite (red). FTIR spectra displayed the unique chemical bond vibrational characteristics at specific frequencies for each of the above two materials (indicated by arrows) when stimulated with an infrared (IR) source. Inset shows the water contact angles before and after passivation where surface properties were maintained for up to 8 weeks.

The FTIR-ATR fingerprint spectra of the soft, brain-like composite is compared to that of polydimethylsiloxane (PDMS) in Figure 3. In general, for PDMS (Sylgard 184), the characteristic peaks were found at 796, 844, 865, 1018, 1078, 1259, 2356, 2964 and 3263 cm^−1^, similar to prior reports [59]. Different absorbance levels were observed at 2356 cm^−1^ (-Si-H), 2962 cm^−1^ (Si-CH_3_) and 865, 844 cm^-1^ (Si-OH) for varying degrees of crosslinking that produced a range of elastic moduli (as shown earlier in Figure 1). Nanotube incorporation into the silicone network results in modulation of peaks at 1018:1078 cm^−1^ and the addition of a D-band peak at 1245 cm^−1^, G-band and D-band peaks at ~1640 and 1420 cm^−1^ regions, respectively. However, there is some overlap with the PDMS FTIR spectrum for 1300–1800 cm^−1^ regions with vinyl groups of unreacted crosslinker. The passivation step facilitated the removal of unreacted crosslinkers and exposed the G-Band and D-Band regions of nanotubes. In addition, passivation modified the material on the surface with -OH groups (peak at 3411 cm^−1^) and decreases the -Si-CH_2_-Si peak at 1078 cm^−1^. This addition of -OH groups increased the overall surface energy, resulting in lower water contact angles as observed in the inset in Figure 3. Surface passivation resulted in a decrease in water contact angle from 97–103° to 59–64°. The surface properties were maintained in stability tests where passivated soft brain-like composites that were immersed in aCSF for 8 weeks had water contact angles of 48–51°. Furthermore, gravimetric tests for swelling showed <1% change after 8 weeks of immersion in aCSF (data not shown). The bulk resistivity (ρ) of the final crosslinked brain-like coating was estimated to be 31.0–41.3 kΩ-cm. Overall, the synergistic combinations of (a) low crosslinking of PDMS resulting in low elastic modulus, (b) the decrease in exposure of hydrophobic group (-CH_3_) and (c) the increased exposure of silanol groups (Si-OH) due nanotube incorporation and surface passivation treatment allows for a highly biocompatible and novel material with flexible functionality for interfacing bioimplants.

### 3.2. Primary Cortical Neurons Show Higher Viability on Soft Substrate in In Vitro Tests

**Figure 4:**
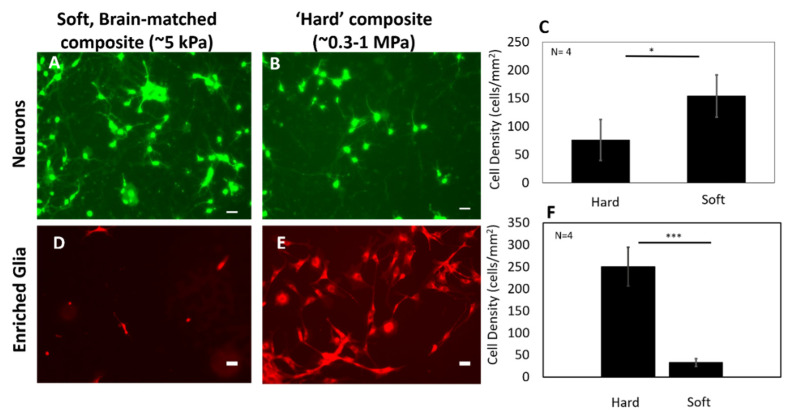
Comparison of cell proliferation and morphology of (**A**–**C**) cultured rat cortical neurons (DIV 7) and (**D**–**F**) enriched primary glial cultures (cultivated for ~36 h prior to imaging) from rat cortex cultivated on (**A**,**D**) hard silicone substrates (*n* = 4 each condition) (0.3–1 MPa) and (**B**,**E**) soft coated silicone substrates (*n* = 4 each condition) (5–10 kPa). (**C**) The neuron cell viability imaged using Calcein AM on soft substrates was significantly higher (* *p* < 0.05) compared to ‘hard’ composite substrates (right), where soft substrates had approximately 154 ± 37 cells/mm^2^ and ‘hard’ substrates had approximately 76 ± 36 cells/mm^2^. (**F**) Cell densities for enriched glia cultures imaged using Mitotracker-CMXRos™ as a live cell marker are significantly increased for harder substrates (250 ± 50 cells/mm^2^) compared to softer substrates (33 ± 17 cells/mm^2^) (*** *p* < 0.001). Bar indicates 50 µm.

Dissociated primary cortical neurons were seeded on soft brain-like silicone composite substrate (E~5 kPa) and on ‘hard’ silicone composite (E~300–1000 kPa) (Figure 4). After 7 DIV, the cells were imaged using live assay. Neurons cultured on the soft brain-like substrates had a significantly higher cell viability (154 ± 37 cells/mm^2^) compared to those on ‘hard’ substrate (76 ± 36 cells/mm^2^) (*p* < 0.05). In addition, neurite morphology was also different. While the majority of cells on both substrates exhibited primary branching, 17.3% of imaged cells on the soft brain-like composite substrates had secondary neurite branching compared to 8.8% of the imaged cells on ‘hard’ substrates. In comparison, enriched glia cells with grown soft brain-like substrates ((33 ± 17 cells/mm^2^) had a significantly lower cell viability compared to those on ‘hard’ substrates (250 ± 50 cells/mm^2^) (*p* < 0.001).

### 3.3. In Vivo Electrical Impedance Is More Stable For Soft-Coated Electrodes

**Figure 5:**
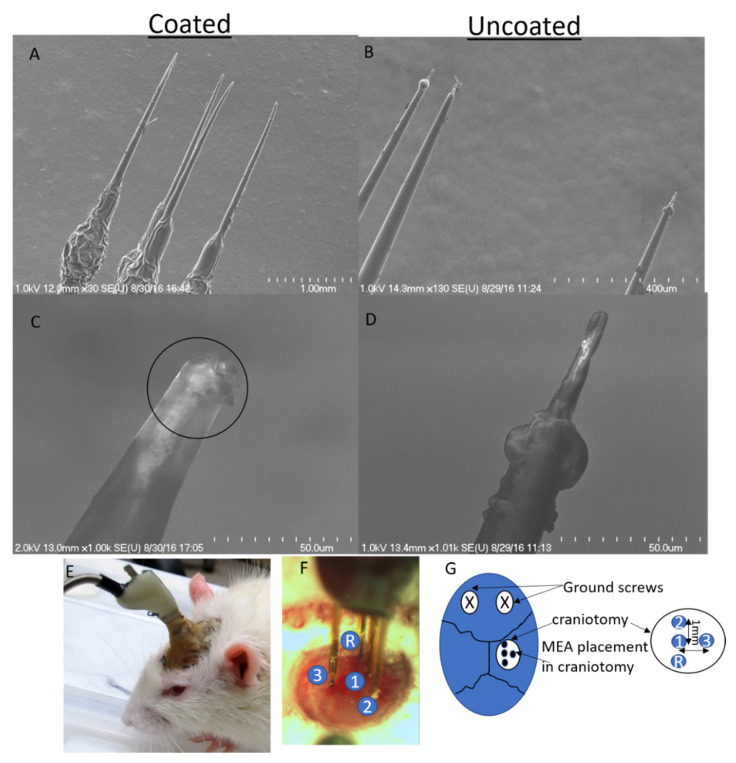
(**A**) Commercially available 3-channel tungsten microarrays (Microprobes, LLC.) with one reference electrode dip-coated with brain-like soft composites. Representative uncoated tips in control arrays are shown in (**B**). Close-up images of a representative dip-coated probe (**C**) show that the recording site (circled) is completely covered with the composite compared to the representative uncoated probes (**D**). Representative image of the implanted array in the rat somatosensory cortex is shown in (**E**,**F**). Relative recording probes sites are marked in (**F**). A schematic showing the relative placement of ground screws and MEA is shown in (**G**).

Three-channel commercial tungsten microwire (125 µm diameter) arrays were coated with the soft brain-like silicone/CNT composite at the recording site and implanted in rats. Representative examples of field emission scanning electron micrographs (FESEMs) of soft-coated arrays are shown in Figure 5. A total of 6 uncoated tungsten electrodes were used as negative controls and a total of 9 coated tungsten electrodes were tested in five different animals. Overall, two animals had mixed arrays with both coated and uncoated electrodes, while one animal had an array with electrodes that were not coated and two animals had all their electrodes coated with the soft silicone composite. (See Table 1).

**Figure 6:**
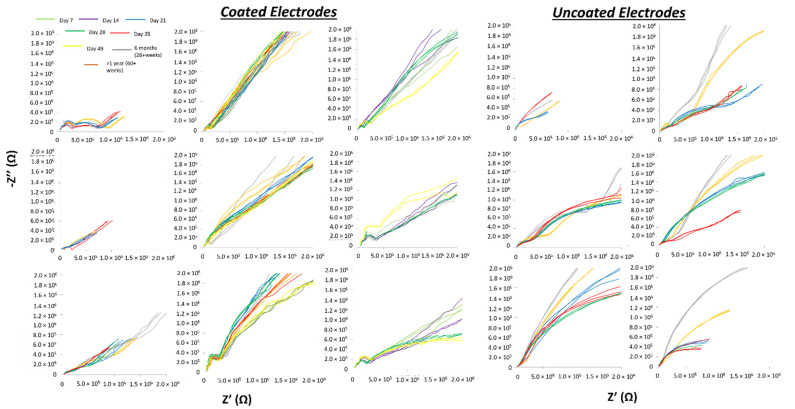
Nyquist plots of coated (*n* = 9 electrodes) and uncoated electrodes (*n* = 6) from 5 animals are shown over 5–7 weeks of implantation. Soft-coated electrodes show relative stability in electrical impedance after the first week over long implantation periods compared to 4 of 6 uncoated electrodes.

Complex broad-band impedance spectra showing electrical impedance over a range of frequencies were plotted as Nyquist diagrams of long-term experiments and are shown in Figure 6. Qualitative assessments showed that uncoated electrodes became more resistive with longer implantation times as observed by a generally decreasing slope trending towards the real axis (resistance) over 5–7 weeks in four of the six uncoated electrodes. The other two of the six uncoated electrodes maintained their slopes in Nyquist plots over 5–7 weeks of implantation. In contrast, the majority of soft-coated electrodes maintained their slopes in Nyquist plots over long implantation periods over 5–7 weeks.

**Figure 7:**
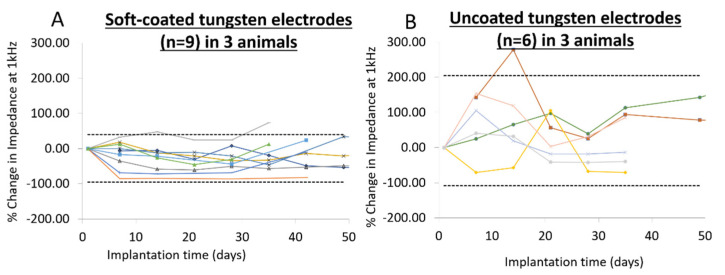
Comparison of change in absolute impedance at 1 kHz for (**A**) coated and (**B**) uncoated tungsten electrode over 5–7 weeks. Dotted lines indicate 95% confidence intervals assessed over all data points across implantation times within the group (coated vs. uncoated). Tungsten electrodes coated with soft siloxane were relatively more stable over implantation time compared to uncoated controls.

In order to better capture the relative changes in impedance, the percent change in impedance at 1 kHz over a duration of 5–7 weeks for coated and uncoated controls is shown in Figure 7. Four of six uncoated electrodes reached their peak impedance by 14 days of implantation and all six uncoated electrodes experienced an increase in impedance within 21 days of implantation. Overall, at its peak, uncoated electrodes experience +105–280% change in impedance at 1 kHz. At the end of 5–7 weeks, electrical impedance of the other three uncoated electrodes decreased back to values closer to those on day 1. In contrast, electrical impedance of only one of nine soft-coated electrodes reached a peak increase of 47% in the first 21 days of implantation, while eight of nine electrodes were generally stable and experienced low levels of fluctuation in electrical impedance. After 5–7 weeks, electrical impedance of eight of nine electrodes remained within ±33% of their initial impedance value (95% confidence interval), while the electrical impedance of the other one of nine soft-coated electrodes had a +74% change.

Overall, the average initial (day 1) electrical impedance at 1 kHz for uncoated electrode (*n* = 6) was 1.72 × 10^5^ ± 1.54 × 10^5^ ohms and 1.69 × 10^6^ ± 1.91 × 10^6^ ohms for soft-coated electrodes (*n* = 9). The median for soft-coated electrodes was 6.2 × 10^5^ ohms compared to 1.3 × 10^5^ ohms for uncoated controls. The larger range for soft-coated electrodes could be due to variation in coating thickness or nanotube distribution or differences in surface passivation. The measured impedance fluctuated as implantation time increased over 5–7 weeks.

**Figure 8:**
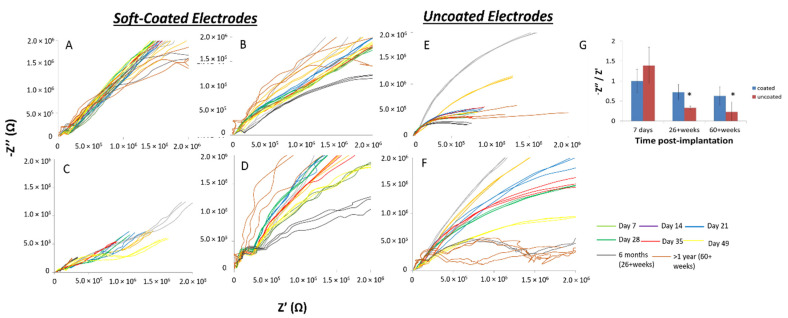
Broad-band complex impedance spectra for coated (**A**–**D**) and uncoated (**E**,**F**) tungsten electrodes over > 432 days (60+ weeks). Uncoated electrodes become more resistive (slope decreases) with longer implantation times. Soft-coated electrodes in (**A**–**D**) maintain similar slopes over long-term implantation. The soft-coated electrode in (**D**) shows more variability with > 6 months (+26 weeks) data having a more resistive slope compared to >1 year (60+ weeks) having a more capacitive slope. (**G**) shows the comparison of the slopes from a linear fit of averaged complex impedance spectra (−Z″/Z′) for soft-coated and uncoated electrodes. The values for the uncoated electrodes decreased significantly (* *p* < 0.05), which is indicative of the impedance becoming more resistive with longer implantation times.

In a subset of rats (two animals), a total of *n* = 2 uncoated electrodes and *n* = 4 soft-coated electrodes were implanted for >1 year (Figure 8). Overall, uncoated electrodes (Figure 8E,F) became more resistive compared to soft-coated electrodes which maintained their slopes. The Nyquist plot of the soft coated electrode in Figure 8D showed more variability within the first 6 months (26+ weeks), possessing a more resistive slope compared to the one at >1 year (60+ weeks), which had a steeper slope. Linear fits of the averaged complex impedance spectra showed that the slopes (-z”/z’) for uncoated electrodes decreased significantly (*p* < 0.05), which is indicative of the spectra becoming more resistive with longer implantation times (Figure 8G). Soft-coated probes showed no statistically significant change with long implantation times lasting more than 1 year.

**Figure 9:**
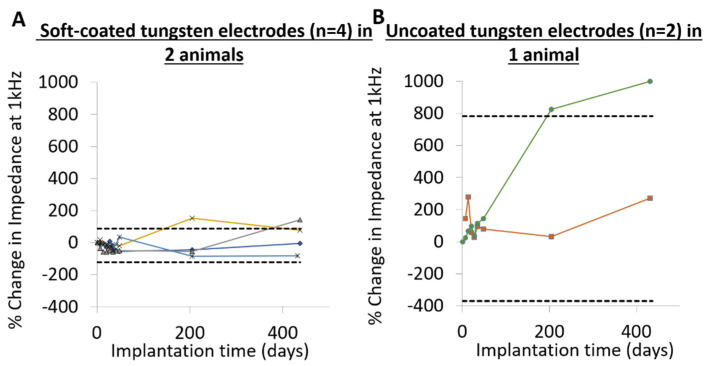
Comparison of change in absolute impedance at 1 kHz for (**A**) soft-coated and (**B**) uncoated tungsten probes over >432 days. Dotted lines indicate 95% confidence intervals assessed over all data points across implantation times within the group (soft-coated vs. uncoated).

In order to compare the impedance at 1 kHz (Figure 9), at the end of ~6 months of implantation, electrical impedance of one uncoated electrode increased to 825% of day 1 starting values, while the impedance of the second uncoated electrode increased by a more modest +30%. By >432 days (60+ weeks), impedances of both uncoated electrodes increased further, with one ~+1000% more and the other +272% of original values. In contrast, at the end of approximately 6 months of implantation, three of four soft-coated electrodes maintain or had lower impedance, while impedance of one of four electrodes increased to +150% of original impedance value. By >432 days (60+ weeks), two of four soft coated electrodes maintained or had lower impedances compared to day 1, while electrical impedance of the other two increased to +87% and +150% of day 1 values.

**Figure 10:**
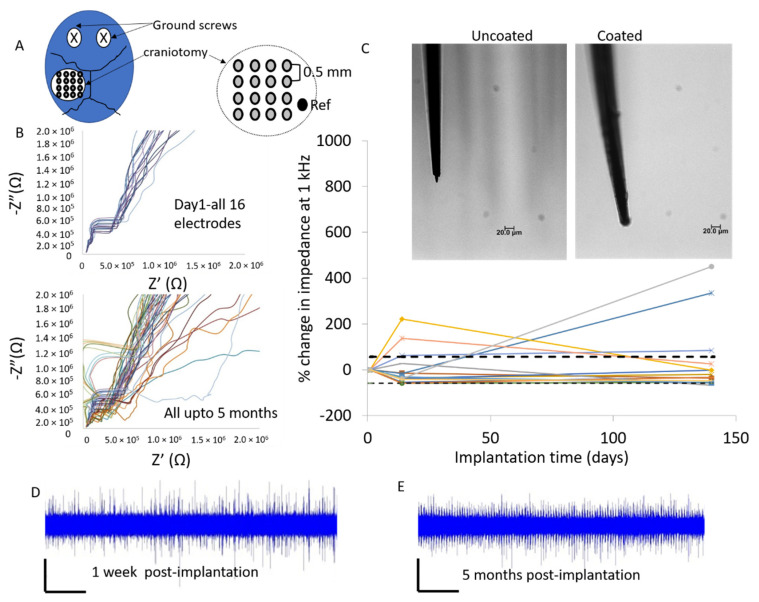
(**A**) Schematic of the 16 channel Pt/Ir MEA placement in the somatosensory region of the cortex is illustrated. (**B**) Nyquist plots of all 16 channels of Pt/Ir array implanted in the somatosensory cortex showing relatively stable trends over 5 months. (**C**) Comparison of impedance at 1 kHz show 12 of 16 electrodes having relatively stable impedance. Inset shows representative light microscope images of soft-coated and uncoated electrodes. Raw traces of a representative probe with soft coating at the tip after 1 week of implantation (**D**) and 5 months of implantation (**E**) are shown. Vertical bar is 50 µV and horizontal bar is 10 s.

In the case of the 16-channel soft-coated Pt/Ir microwire arrays, electrodes were implanted in one animal in the somatosensory region of which five electrodes in the array were placed in the barrel cortex region. The broad-band EIS of the coated Pt/Ir in vivo is plotted in Figure 10 over 5 months of implantation. Seventy-five percent (12 of 16) of the soft-coated Pt/Ir electrodes had relatively stable impedances over 5 months of implantation. Two of sixteen electrodes showed increased (>+150%) after 2 weeks, while two other electrodes showed +335% and +450% increase after 5 months. In all four electrodes where impedance at 1 kHz increased >150%, an increase of >50% in noise levels was also observed after 5 months. The average impedances across all 16 electrodes at 1 kHz post-implantation were 3.8 ± 1.1 MΩ (day 1), 3.8 ± 3.3 MΩ (after 2 weeks) and 4.9 ± 5.6 MΩ (after 5 months). The higher averages and standard deviations were due to the contribution of the four electrodes with high impedances observed in Figure 10. With these four points excluded, the average impedances at 1 kHz post-implantation were 2.4 ± 0.8 MΩ (after 2 weeks) and 1.3 ± 0.4 MΩ (after 5 months). The average noise levels for all 16 electrodes were 23.7 ± 3.6 µV (day 1). At 5 months, the noise levels for 12 of 16 electrodes with stable impedances were 29.6 ± 16.8 µV and for 4 of 16 electrodes with high impedances were 61.1 ± 15.2 µV. Beyond 5 months, the animal was euthanized due to issues unrelated to the implant.

### 3.4. Peak-to-Peak Amplitudes for Isolated Single Units Are Larger for Soft-Coated Electrodes

**Figure 11:**
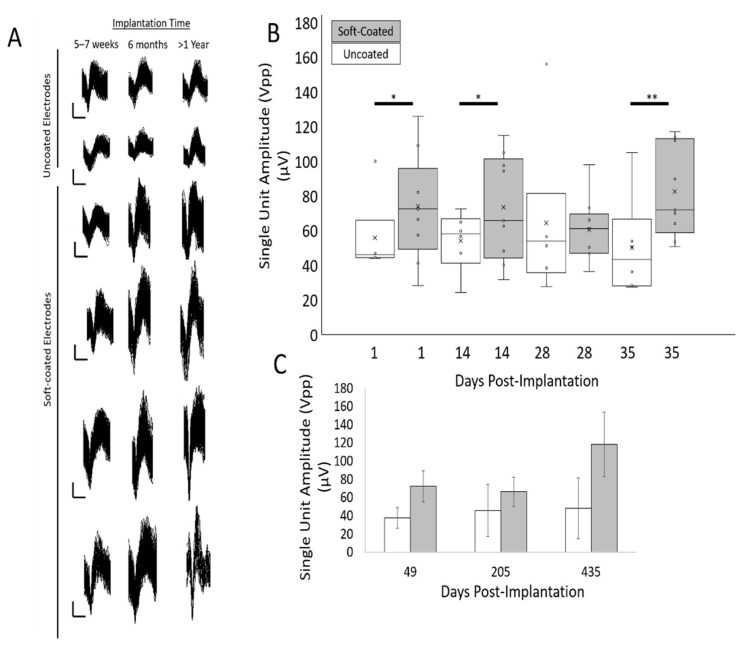
(**A**) Representative examples of single unit waveforms isolated from chronic neural recordings of spontaneous neural activity using tungsten microelectrodes from 5–7 weeks to >1 year. Vertical scale bar is 20 µV and horizontal scale bar is 1 ms. (**B**) The mean peak-to-peak amplitudes (Vpp) of the largest isolated unit recorded from spontaneous neural activity is plotted for soft-coated (*n* = 9) and uncoated electrodes (*n* = 6) over 5 weeks of implantation. * *p* < 0.05; ** *p* < 0.01. (**C**) Long-term trends for mean peak-to-peak amplitudes (Vpp) of the largest isolated unit recorded from spontaneous neural activity are plotted for soft-coated (*n* = 4) and uncoated electrodes (*n* = 2) over 1 year of implantation. Soft-coated electrodes trended toward having larger peak-to-peak amplitudes compared to uncoated controls.

**Figure 12:**
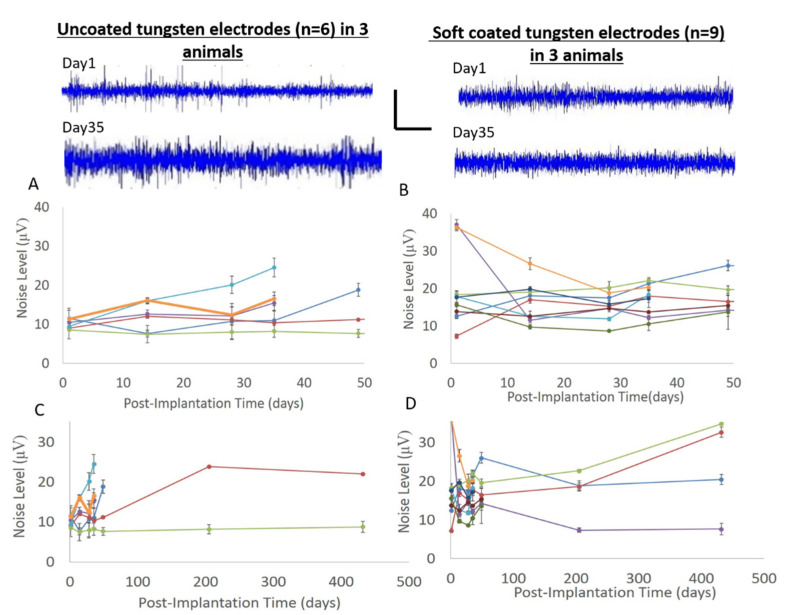
Noise levels are compared for uncoated tungsten in (**A,C**) and for soft-coated tungsten in (**B**,**D**). Raw traces from select channels at 1 day post implantation and 35 days (5 weeks) post implantation for uncoated (left) and coated probes (right) show modest increase in noise levels for uncoated probes, while soft-coated probes typically did not change. Vertical bar is 50 µV. Horizontal bar is 0.1 s. (**C**,**D**) show that long-term trends for uncoated and coated probes, respectively remain between 10 and 30 µV.

**Figure 13:**
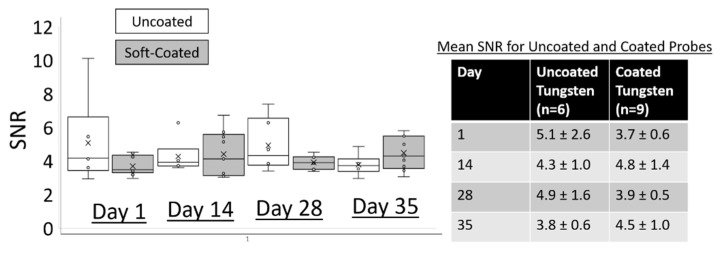
Comparison of SNR for uncoated (white) and coated (gray) tungsten electrodes show similar range of values across post implantation timepoints.

Finally, neural recordings from tungsten electrodes were taken at different time points over 60+ weeks and assessed for signal quality. The maximum peak-to-peak amplitudes among the sorted single units over a duration lasting > 1 year in both uncoated and coated tungsten electrodes are shown in Figure 11. The maximum peak-to-peak amplitudes in the uncoated tungsten electrodes did not reach an excess off 60 µV, while those from soft-coated tungsten electrodes were in the range of 60–150 µV. The mean number of units decreased ~45% from 3.7 ± 0.4 to 1.7 ± 1.0 by 5–7 weeks for electrodes with uncoated tips (*n* = 6), while the mean number of units for electrodes with coated tips (*n* = 9) increased ~63% from 2.7 ± 0.9 to 4.4 ± 0.7 by 5–7 weeks as seen in Table 2. At longer timepoints (>1 year), discernable units in electrodes with uncoated tips are reduced to 1 unit (*n* = 2 electrodes), while electrodes with coated tips maintained a mean of 4 units (*n* = 4 probe tips). Typical noise levels ranged from 8–25 µV for uncoated tungsten probes for all timepoints as seen in Figure 12A,C. The range of noise levels for coated probes were larger between 8 and 38 µV for all timepoints with the majority of data between 10 and 20 µV, as seen in Figure 12B,D. Comparison of SNR for uncoated and coated tungsten electrodes show a similar range of values with average median SNR values of 4.0 ± 0.26 (uncoated probes) and 3.9 ± 0.31 (coated probes) across all post implantation timepoints (Figure 13).

**Figure 14:**
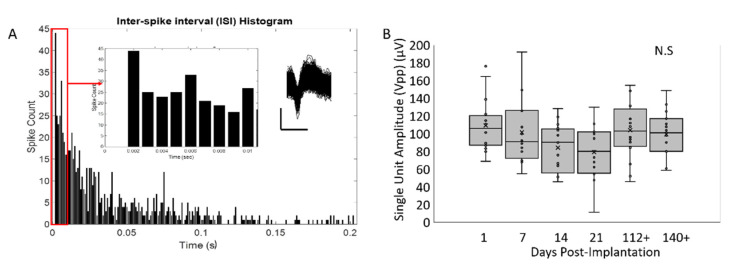
(**A**) ISI histogram of a single unit isolated from spontaneous neural activity after 5 months of implantation using a 16-electrode, Pt/Ir array. Bin size is 1 ms. Inset on the left shows a closeup of the histogram indicating no spikes in the refractory period. Inset on the right shows representative single unit waveforms isolated from a 2 min block of neural recordings. Vertical bar is 50 µV and horizontal bar is 1 ms. (**B**) Plot of Vpp vs. implantation time for all 16 electrodes (Pt/Ir array) over 5 months shows no significant differences (N.S) in the largest single unit isolated from spontaneous activity in all 16 electrodes. For 3 week and 5 month time points, single units were not isolated. Mean is indicated with ‘×’ and individual electrode values are indicated by ‘°’. Significance was assessed using one-way ANOVA (α~0.05).

The peak-to-peak amplitudes of the largest single unit from spontaneous activity from the soft-coated, platinum–iridium electrodes are shown in Figure 14. A representative waveform with its associated inter spike interval (ISI) histogram is plotted in Figure 14A. Typical sorted units resulting from spontaneous activity had ~80–100 µV in peak-to-peak amplitudes after 5 months. While the spontaneous neural recordings were predominantly conducted in passive situations, some soft-coated Pt/Ir electrodes were implanted in the barrel cortex. Single and multi-unit responses to whisker stimulation with peak-to-peak amplitudes upwards of 150–250 µV were observed in five of the soft-coated Pt/Ir electrodes implanted in the barrel region (data not shown).

## 4. Discussion

In this study we developed a soft brain-like, silicone/CNT based composite that made an excellent candidate for mimicking brain tissue material properties. Off-the-shelf silicone-based materials incorporated with CNTs were tuned to an elastic modulus of ~5 kPa similar to rat cortical brain by varying the levels of cross-linking (Figure 1). While prior studies on development of soft neural interfaces have so far attempted to fabricate materials to match the elastic modulus of brain tissue. To our knowledge, no attempt has been made to match the relaxation time constants of brain tissue typical of a viscoelastic material. Under small strains, both the brain tissue and the silicone/CNT composite reported here resemble a Maxwell type model with time constants for stress relaxation that correspond to a fast phase followed by a slow phase. In this current study, viscoelastic relaxation properties of brain tissue were matched by tuning the fast relaxation phase of the soft silicone composite to match that of brain tissue. The elastomeric interface matched the viscoelastic properties of rodent brain in vivo better than agarose as summarized by the force-displacement curves and the estimated time-constant values in Figure 2. A step-indentation methodology (previously established in [49]) was utilized to measure and match the viscoelastic properties of in vivo cortical tissue and silicone/composite materials (see methods). As shown in Figure 2C, the median time constant for the fast relaxation phase of the brain tissue (13.3 s) closely matched the soft silicone composite (17.2 s). Both of the above time constants were significantly larger than the median time constant of 0.5% agarose gel (5.99 s) that is commonly used as a model of brain tissue. Longer relaxation time constants imply lower strain and strain rates, potentially resulting in reduced micromotion-induced long-term injury [49]. Previous studies also showed that soft probes composed of cellulose-based nanocomposites (with elastic modulus ~12 MPa) had reduced strain rates compared to silicon-based probes [42]. In addition, these probes decrease the micromotion induced by mechanical stresses (implicated in generating chronic mechanical strain) that potentially results in reduced interfacial immune response [42,60]. Similar observations were reported for thioacrylate based ‘softening’ probes, where histology showed improved biocompatibility compared to parylene-coated electrodes [61]. Therefore, lowering strain rates seems to be an important factor among other factors such as shape and geometry for improving the long-term stability of neural interfaces.

Soft brain-like silicone/CNT composites are conducive to the growth of cortical neurons (DIV 7) with increased viability and neurite differentiation exhibited by increased branching complexity (Figure 4). In addition, the soft brain-like composite surface is less conducive to glial proliferation. Several studies that examine neuronal stem cell maturation and differentiation suggest that the morphology of neurons increase in complexity with decreased substrate stiffness [62,63,64]. Harris et al. has shown that manipulating the mechanical microenvironment (i.e., stiffness properties of the substrates) improves the density of neuronal populations near the implant [65]. In addition to providing a conductive path for obtaining neural recordings, previous work suggests that the incorporation of CNTs contributes toward the improvement of neuronal differentiation in culture [66]. Therefore, the synergy of a mechanically soft brain-like substrate and CNTs is expected to improve the local microenvironment near the interface with increased neuron populations. However, the question remains whether the stability of electrical conduction properties under long-term implant conditions improve due to modulation of stiffness properties at the interface.

Due to variations in thickness or relative carbon nanotube distribution or changes in surface passivation at the recording site, the addition of the soft brain-like coating increases the impedance at 1 kHz to ~ 5 × 10^6^ Ω, which is still within the acceptable range for recording electrodes. For soft-coated, platinum–iridium electrodes, the impedance at 1 kHz increases to ~3.8 × 10^6^ Ω. While the dip-coated thickness of the interface ranges from 20–40 µm, it was not optimized and is beyond the scope of this study for which the main aim is to determine whether soft brain-like interfaces improved electrical characteristics. Future studies will focus on the optimization of the coating thickness, nanoscale distribution and characterization of carbon nanotubes in relation to the relative quality of the neural recordings.

Electrical impedance at 1 kHz is a key performance metric that is widely used to assess the integrity of neural interfaces and its ability to record electrical activity from single or multiple neurons that may be in the vicinity. So far, achieving a stable electrical impedance at the neural interface over long durations of implantation has been an elusive target that has contributed to the unreliable performance of these neural interfaces. In this report we showed that the soft silicone composites had stable surface passivation properties over 8 weeks in a CSF, possibly due to silanol groups that are brought to the surface to minimize surface energies by hydrophilization and by delaying the hydrophobic recovery process [67,68]. It should be noted that the elastic modulus of the soft silicone composite was not significantly different after 4 weeks in aCSF, suggesting stability in their mechanical properties under simulated body-conditions.

The primary goal was to test the hypothesis that soft mechanically matched neural interfaces at the tips of microelectrodes improve the electrical characteristics and functionality of neural implants. A key result of this study is that correcting mechanical mismatch at the recording site located at the tip using a ‘brain-like’ soft coating on the implant stabilized electrical impedance of the interface for implantation durations lasting over 1 year (Figure 6, Figure 7, Figure 8, Figure 9 and Figure 10). The relatively stable, electrical interface is also observed with minimal changes in the complex broad-band impedance spectra plotted as Nyquist diagrams for coated tungsten electrodes after 5–7 weeks of implantation. In comparison, four of six uncoated tungsten electrodes became increasingly resistive after 5–7 weeks of implantation. Previous studies using similar uncoated tungsten microwire arrays showed drastic changes in impedance (>+600%) at 1 kHz over 5–7 weeks [9], consistent with the results observed for uncoated controls in this current study. In addition, three of four coated tungsten electrodes maintained low impedances over 6 months of implantation and two of four coated tungsten electrodes maintained low impedances over 432 days. Soft-coated platinum–iridium electrodes also maintained low impedances in 12 of 16 electrodes over 5 months of implantation. The soft brain-like coating therefore stabilizes electrical impedance at 1 kHz at the neural interface in vivo. It should be noted that the point of extending the study to Pt/Ir electrodes was only to test if the coating process and adhesion stability of siloxane and any accompanied improvement in stability of electrical impedance was also achieved in other electrode systems such as Pt/Ir under chronic conditions.

**Figure 15:**
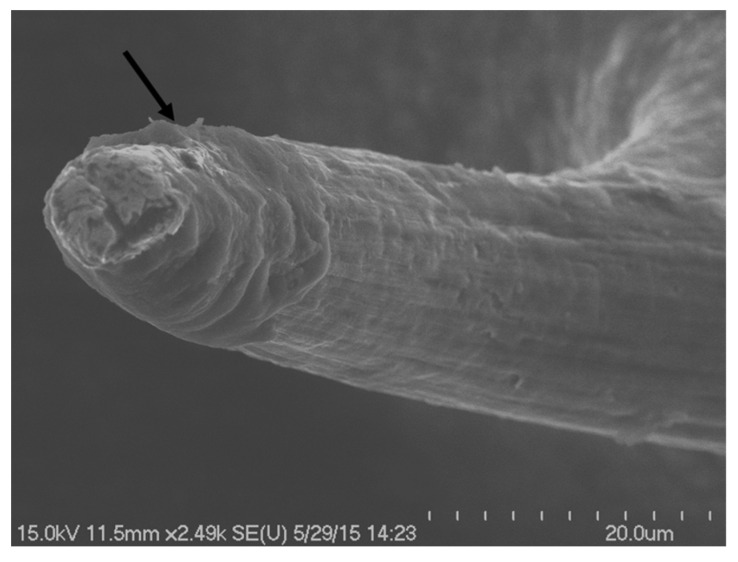
Postmortem image of a coated tungsten probe after >1 year of implantation. An intact probe tip with the coating (arrow) is shown.

The changes in uncoated electrodes followed similar trends in Nyquist plots that have been reported in many prior studies. Williams et al. notes that the shifts in complex impedance spectra could be correlated to the tissue encapsulation process [69]. Sankar et al. speculates that the changes in the complex impedance spectra are possibly due to a combination of multiple processes such as the inflammatory response at the interface, corrosion and insulation deterioration at the electrode interface [9]. Kozai et al. examined numerous cases of material failure and glial encapsulation that results in similar observations of changing impedance spectra with chronic implantation time [44]. The relatively stable Nyquist plots for the soft brain-like electrodes in this study therefore suggests a stable milieu at the interface where both biotic and abiotic changes are minimal corresponding to minimal tissue reaction/glial scarring and minimal corrosion and electrode degradation. In this study, FESEM based imaging of soft-coated electrodes post >1 year of implantation showed no discernable pitting or corrosion in the underlying tungsten. No visible loss or degradation of the soft coating was observed in long-term studies (Figure 15). The long-term electrical performance of the soft brain-like composite showed increased amplitudes of single units and increased number of detectable single units in spontaneous neural recordings obtained under chronic implantation conditions. Single units sorted from neural recordings obtained from soft-coated electrodes at 5–7 weeks, 6 months and 1 year had a higher peak-to-peak amplitude (60–150 µV) compared to uncoated tungsten controls (<60 µV). Similar trends were observed in soft-coated Pt/Ir electrodes which typically had isolated units with ~100 µV amplitudes after 5 months of implantation. Decreasing amplitude changes in action potentials have been observed in other studies over implantation time and were associated as a potential factor for device reliability [70]. McCreery et al. [71] suggests that the largest action potential amplitudes were better correlated with neuron density and tissue health (measured by GFAP intensity) nearest to the probe (<80 µm) compared SNR, which correlated better to regions further from the interface. Therefore, the relative increase in the amplitude and number of detected units can be indicative of the tissue quality and probe stability at the interface. In this study, we speculate that the increasing amplitude of units near soft-coated probes correspond either to lesser numbers of silent neurons at the neural interface or a higher neuron density compared to uncoated probes, which is corroborated by an increase in the mean number of units detectable at the interface (Table 2). However, it should be noted that electrical characteristics, such as unit amplitude, are highly dependent on multiple factors such as neuronal type, relative electrode position and stimuli. It is evident that soft-coated electrodes implanted in the barrel cortex recorded unit activity upwards of 200 µV in amplitude. Ironically, metrics assessing long-term signal quality in terms of signal-to-noise (SNR) (calculated by using either the method by Ludwig et al. [58] or Jackson et al. [72]) were more conservative compared to the apparent increase in peak-to-peak voltages (Vpp) for both soft-coated and uncoated electrodes possibly due to shifting noise levels at different timepoints. This could be due to the fact that SNR values are determined by numerous factors, such as the presence of silent neurons, conscious state and spontaneous activity level of the animals during recording sessions and target neuron-electrode distance, etc. Chronic shifting changes in SNR at different electrode sites have been previously documented [73].

## 5. Conclusions

An elastomeric soft silicone/CNT composite was developed that matched the viscoelastic properties of brain tissue (by matching both the elastic modulus and the relaxation time constant). The elastic modulus of the soft silicone composite was tunable by varying the degree of cross-linking. The dip-coating fabrication method and the materials used in this study are readily adoptable by laboratories across the world for a range of existing microelectrode arrays. The EIS of soft silicone composite neural interface at the microelectrode tip was tested using both conventional tungsten and Pt/Ir electrodes in long-term experiments lasting from 5 months to over 1 year and was found to be relatively more stable than the EIS of the uncoated conventional uncoated interfaces. The soft brain-like coatings also improved the number of discernible units and the peak-to-peak amplitudes of single units detected in the spontaneous neural activity.

## Figures and Tables

**Table 1:** Experimental details for cohort of rats in this study (*n* = 6).

Rat#	Days Implanted
#1 (3 uncoated tungsten electrodes)	35
#2 (3 coated tungsten electrodes)	437
#3 (1 coated, 2 uncoated tungsten electrodes)	432
#4 (2 coated, 1 uncoated tungsten electrodes)	35
#5 (3 coated tungsten electrodes)	49
#6 (16 coated, platinum–iridium electrodes)	140

**Table 2:** Number of isolated units across implantation time for tungsten microelectrodes with uncoated and coated tips.

Timepoint (Day)	Uncoated Tungsten (Mean No. of Units)	Coated Tungsten (Mean No. of Units)
1	3.7 ± 0.4	2.7 ± 0.9
14	2.7 ± 0.5	3.3 ± 0.5
28	2.5 ± 1.0	3.8 ± 0.7
35	1.7 ± 1.0	4.4 ± 0.7
49	2.0 ± 1.4	4.3 ± 0.5
180+	1.5 ± 0.7	3.3 ± 0.5
>1 year	1 ± 0	4.0 ± 0.8

## Data Availability

Data are available from the authors upon request.
